# The Nitrie of Amyl

**Published:** 1870-07

**Authors:** 


### Selections.

---

The Nitrite of Amyl —At a meeting of the Clinical Society of London, in February, Dr. Anstie communicated a case of angina pectoris relieved by nitrite of amyl. The patient was a gentleman aged about 50, of a highly nervous temperament, a sufferer for the last twenty years from spasmodic asthma, and very liable to facial neuralgia. Between four and five years since he began to suffer from severe and frequently recurring attacks of angina pectoris, and his life was for a long time in much danger. By the use of sulphuric ether in large doses, and of considerable quantities of alcoholic stimulants, the attacks were diminished in frequency and violence; but they have never ceased to recur at intervals whenever the patient was much fatigued or excited in the course of his professional work. In December last it was determined to try the nitrite of amyl, and on the recurrence of the next anginal spasms, the sufferer took one long and powerful inspiration through one nostril from a half-ounce bottle of the drug. After a pause of a few seconds, the characteristic flushing of the face and sense of fulness in the head were induced, and the patient instantly passed from agony into a state of perfect calm repose.

The experiment has been several times repeated on the recurrence of the heart-pang, and always with complete success, and the result has been most fortunate, as the patient has been able to entirely dispense with the disagreeable necessity of taking large and frequent doses of ether, and also greatly to reduce his allowance of stimulants. There has also been much less trouble than formerly from asthma, and he has obtained far more natural sleep at nights. The angina, although very severe, appears to be purely neurotic; no cardiac change, or at most a small amount of dilatation can be discovered by the most minute physical examination. From some further observations Dr. Anstie believes that amyl is a relaxer of spasm in all involuntary muscular fibre, and it would be particularly useful in colicky affections. He would not, however, advise its use by aged persons or others who might be likely to have commencing degeneration of the minute vessels of the brain, for fear of appoplectic accidents.

Dr. Farquhar said that in India he had seen a gentleman suffering from a kind of colic, paroxysmal in its character, and coming on at night very terrible to the sufferer. Bromide for a time gave relief. He then tried the nitrite of amyl, when, as the patient expressed it, he was transferred from agony to heaven in a moment. The disease partially returned when the drug was discontinued. In another instance, similar to the above, the nitrite also did good. In reply to Dr. Buzzard, he stated that the attacks seemed to wear out.

